# Awakening, condensation, and sedimentation: the correlation mechanism between topics and emotions in online public opinion from the psychosocial anchoring perspective

**DOI:** 10.3389/fpsyg.2025.1624689

**Published:** 2025-08-18

**Authors:** Hong Yu, Menglan Ma, Xiangrong Yu, Peng Fang

**Affiliations:** ^1^School of Journalism and Information Communication, Huazhong University of Science and Technology, Wuhan, China; ^2^School of Computer Science and Technology, Huazhong University of Science and Technology, Wuhan, China

**Keywords:** online public opinion, food safety crises, social representation theory, psychosocial anchoring, topics-emotions dynamics

## Abstract

Emotions significantly shape how publics interpret and engage with crises on social media, yet most public-opinion research treats thematic structures and affective dynamics in isolation. Drawing on social representation theory and psychosocial anchoring, this mixed-methods study examines the interplay of topics and emotions during the 2023 “Rat Head and Duck Neck” food safety incident on Chinese social media. By analyzing posts through LDA topic modeling, TextCNN-based sentiment analysis, and qualitative content analysis, the analysis reveals three dominant emotions: disgust, anger, and satire, and three central topics: food, trust, and power. These elements co-evolve via three interaction patterns: awakening, condensation, and sedimentation. These patterns not only map the evolution of online discourse but also reveal psychosocial mechanisms critical for designing more emotionally attuned digital risk-communication and management strategies in food safety crises.

## 1 Introduction

In recent years, global emergencies have become more frequent, severe, and widespread, posing significant threats to human life and property (McGlade et al., 2019; Sohrabi et al., 2020; Agache et al., 2022). Among these, food safety issues (Henderson et al., 2014) have resonated particularly with the public (Mikulsen and Diduck, 2013). Taking China as an example, earlier incidents such as “Melamine Contamination Crisis” (Gao et al., 2012), “Gutter Oil” (Li et al., 2017), and “Lean Meat Powder” (Di et al., 2019), as well as more recent cases like “Pit Pickled Cabbage” (Li, 2024) and the “Rat Head and Duck Neck” (Liu et al., 2024), have left lasting psychological scars on food consumers. Meanwhile, the rapid development of new media technologies has created an environment conducive to the fermentation, escalation, and eruption of online public opinion during emergencies (Civelek et al., 2016; Saroj and Pal, 2020). The public has increasingly voiced their opinions, attitudes, and emotions on current events, with social media emerging as a key platform for disseminating online public opinion during crises (Jin et al., 2014). As a result, understanding the generation and spread of online public opinion in such contexts has become a common concern among scholars in fields such as journalism (McGregor, 2019), social psychology (Yin et al., 2019), and computer science (Xing et al., 2022).

During the formation, development, and decline of online public opinion in emergencies, the openness, anonymity, and connectivity of the Internet allow the public to engage in more open and bold exchanges of opinions and emotional expression on social media. This dissemination process is shaped by the flow of information (Liu et al., 2019; Zhang and Shi, 2022) and is further driven by the flow of emotions (Liu et al., 2015; Feng et al., 2021). Existing studies have extensively examined the evolution of information and emotional flows in online public opinion during emergencies. Research on information flow in emergency-related online public opinion typically employs methods such as LDA topic modeling (Jelodar et al., 2019), clustering analysis (Romesburg, 2004), and statistical analysis (Dixon and Massey, 1951) to dynamically track global topics over specific periods on social media, aiming to understand topic evolution and identify prominent issues at different stages (Cao and Yue, 2020). As the structure of internet communication evolves, emotions have emerged as a new driving force and trigger in the dissemination and evolution of online public opinion during emergencies. Researchers have used emotion lexicons and machine learning techniques for quantitative emotion analysis, exploring transmission patterns and evolution models (Liu et al., 2015), identifying key nodes in emotional evolution (Zhang, 2016), and proposing strategies for managing and responding to negative emotions (Feng et al., 2021).

However, current research on online public opinion during emergencies still faces several limitations. One key issue is the lack of interdisciplinary integration. While scholars from fields such as journalism, public administration, social psychology, and computer science have approached the topic from their respective perspectives, a “gap” remains between disciplines. As a result, quantitative studies often stay at a descriptive level and lack sufficient theoretical foundation. Furthermore, research on information flow and emotional flow remains fragmented. Although the role of emotions in online public opinion during emergencies is gaining more attention, the interaction between emotional and informational flows has not been fully explored. In other words, existing studies tend to focus on topic characteristics or emotional analysis in isolation, neglecting a systematic examination of how both factors jointly influence the evolution of public opinion.

To address these challenges, this study examines food safety issues by using the “Rat Head and Duck Neck” incident in China as a case study. Drawing on social representation theory and psychosocial anchoring theory, with emotions as an analytical entry point, the relationship between topics and emotions in online public opinion during emergencies is explored. A text corpus was first constructed by crawling, collecting, and organizing Weibo^1^ posts and comments related to the incident. Subsequently, LDA-based topic modeling (Jelodar et al., 2019) and TextCNN-based emotion analysis (Wang et al., 2023) were applied to examine the composition and evolution of topics and emotions within the online discourse. The integration of topic and emotion analyses further allowed for the identification of specific topics associated with distinct emotional categories and clarified the constructive role of emotions in shaping online public opinion. Finally, based on psychosocial anchoring theory, the correlation mechanism between topics and emotions was summarized, highlighting awakening, condensation, and sedimentation as key features characterizing the interaction process of online public opinion during emergencies.

The innovations of this paper are as follows:

- This paper departs from the traditional approach to emergency-related online public opinion analysis, where the flow of information and emotions are typically examined in isolation. For the first time, it offers a systematic analysis of the interplay between topic features and emotional dynamics.
- The paper also seeks to illuminate the dynamic processes involved in constructing social representations and psychosocial anchoring within online public opinion during emergencies. It explores how emotions, through processes such as awakening, condensation, and sedimentation, evolve into social emotions that shape the public's understanding of the crisis, thereby influencing the formation of social representations.

## 2 Materials and methods

### 2.1 Theoretical framework: emotion-driven psychosocial anchoring and the construction of social representations

This study integrates social representation theory and psychosocial anchoring theory to examine how individuals and groups process unfamiliar or crisis-related events through both cognitive and emotional mechanisms. Social representation theory emphasizes how social groups incorporate novel phenomena into stable systems of meaning through communication and interaction, while psychosocial anchoring theory focuses on how individuals interpret uncertain information based on existing values, beliefs, and attitudes. By combining these perspectives, we propose an integrated framework to explain the co-evolution of emotions and topics in online public opinion during emergencies. Specifically, we explore how emotions drive the anchoring of information and facilitate the emergence of social representations, thereby tracing a dynamic path from emotional activation to symbolic construction.

Originally proposed by Moscovici (2001), social representation theory builds on Durkheim's concept of collective representations to counterbalance the individualistic bias in American psychology. It underscores the social origins of cognition and the role of cultural norms, communicative practices, and value systems in shaping collective understanding (Descombes, 2000; Jodelet, 2011). Building on this foundation, Doise (1985, 1992) introduced psychosocial anchoring theory, which explains how individuals reduce uncertainty by anchoring unfamiliar information to pre-existing cognitive-emotional schemas. This anchoring is not only a cognitive process but also an emotionally charged response shaped by social experience.

While these two theories are often used in parallel, we argue that emotion functions as the critical mediating link between them. Emotion is both an immediate psychological response to external stimuli and a socially embedded construct shaped by cultural experiences. It serves as an internal mechanism for meaning construction and an external tool for public expression and negotiation (Damasio, 1999; Guimelli, 2009). Psychosocial anchoring provides the psychological foundation for emotion-driven categorization, while social representation explains how these emotions circulate, become objectified, and are sedimented at the collective level (Mambet Doue et al., 2020). To capture this transformation, we propose a three-stage emotion-driven mechanism: anchoring–mediation-representation, which explains how individual emotional responses evolve into shared symbolic meanings in the public sphere during crises.

#### 2.1.1 Anchoring: emotional activation and cognitive categorization

In the initial stage, individuals encountering sudden, uncertain events experience primary emotional responses such as anger, disgust, and saddness. These responses are shaped by past experiences and socio-cultural context. According to psychosocial anchoring theory, individuals tend to map new phenomena onto familiar frameworks, such as “regulatory failure” or “government negligence” in the case of food safety incidents, thereby facilitating quick cognitive classification and reducing psychological ambiguity (Doise, 1992; Zhao and Liu, 2020). This process resonates with Höijer's (2011) notion of “emotional anchoring,” whereby past emotional experiences are reactivated to guide new judgments.

#### 2.1.2 Mediation: emotional resonance and communicative amplification

Anchored emotions do not remain private; they are externalized through textual, visual, and symbolic expressions on social media platforms. The decentralized structure and interactivity of digital platforms allow emotional content to spread rapidly through memes, jokes, and satirical language, forming multimodal chains of affective circulation. This process activates what Jodelet (2011) terms the “objectification mechanism” (Sui and Li, 2020; Giordani and Silva, 2021) in social representation: abstract emotions are concretized into symbolic references, such as “toxic food” representing institutional failure. Through repeated interactions, private emotional experiences are transformed into shared interpretive frames that shape the public discourse.

#### 2.1.3 Representation: symbolic sedimentation and meaning construction

As certain emotion-topic pairings gain traction through repeated expression and social endorsement, they gradually sediment into stable social representations. The public's understanding of an event shifts from factual assessment to symbolic and moral interpretation (Norton et al., 2021). For example, the “Melamine Contamination Crisis” scandal in China came to signify not just food contamination but a broader critique of regulatory legitimacy. At this stage, emotional framing achieves symbolic closure, becoming a shared foundation for collective judgment and moral positioning. This reflects the symbolic and socializing functions of social representations (Vidrio, 2019; Piermattéo, 2021), through which emotion-laden symbols acquire cultural meaning and become embedded in group memory and public discourse (Guimelli, 2009).

In summary, this study proposes an “anchoring-mediation-representation” mechanism to explain how emotional responses shape meaning-making at both psychological and societal levels. Emotion acts as a trigger for individual anchoring and a catalyst for collective representation. Psychosocial anchoring theory offers a lens to understand how emotions guide cognitive classification, while social representation theory explains how these emotionally charged interpretations are externalized, shared, and institutionalized. Together, the two frameworks, united by the mediating role of emotion, provide a comprehensive theoretical foundation for analyzing the co-evolution of emotions and topics in online public opinion during emergencies.

### 2.2 Data sources

#### 2.2.1 Case and platform selection

This study selects the “6·1” food safety incident at Jiangxi Industry Polytechnic College (hereafter referred to as the “Rat Head and Duck Neck”) as the research case. The reasons for selecting this event are as follows: First, the core issue of food safety is highly sensitive, directly affecting public health and safety and often sparking strong reactions. Second, the incident generated widespread online discussion, addressing issues such as food safety, student rights, government oversight, and public response. Finally, the event provides valuable insights into how a food safety complaint in a university cafeteria escalated into a national issue, prompting a large-scale investigation. This study uses Sina Weibo as the primary platform for analyzing online public opinion. As one of the most widely used and publicly oriented social media platforms in China, Weibo features high user participation, fast information flow, and open interaction, making it especially suitable for studying topic and emotion dynamics during public emergencies (Wang et al., 2022; Xu et al., 2024). Compared to platforms like WeChat^2^ and Zhihu,^3^ Weibo provides a more active space for spontaneous emotional expression and collective meaning-making. The platform selection aligns with the study's theoretical foundation, which draws on social representation theory and psychosocial anchoring theory. Weibo's open discussion structure facilitates the diffusion of emotionally charged discourse and the formation of shared symbolic meanings. During the “Rat Head and Duck Neck” incident, Weibo became the central site of public engagement. By July 31, 2023, the related topics had received over 1.19 billion views and 157,000 comments, demonstrating their significant societal impact. To address potential limitations of relying on a single platform, we cross-validated the Weibo data using content from other platforms such as authoritative news outlets, WeChat, and Zhihu. While these additional sources were not directly included in the computational analysis to preserve methodological consistency, they were used to triangulate key narratives and detect potential omissions. We also implemented real-time data collection during the incident to reduce the effects of *post-hoc* content filtering.

#### 2.2.2 Data collection and organization

Based on trends observed through Baidu Index,^4^ the public discussions on Weibo surrounding the “Rat Head and Duck Neck” incident spanned ~20 days, from its initial outbreak to gradual decline. This study collected data through web scraping, using keywords such as “duck neck,” “rat head,” and “rat head duck neck” to search for relevant discussions on Weibo related to the incident. The data was collected from 00:00 on June 1, 2023, to 24:00 on June 24, 2023. The collected fields included Weibo content, creation time, number of reposts, comments, likes, comment content, user nicknames, certification type, number of followers, and other related information. After data cleaning and deduplication (removing irrelevant or invalid data, such as posts containing only emojis, links, or advertisements, and filtering out special characters and symbols like emojis and “@XXX” or “#XXX#” without substantive content), a total of 4,208 Weibo posts and 25,191 comments were collected, resulting in 29,398 data samples. The users participating in the Weibo discussions of the “Rat Head and Duck Neck” incident can be divided into three main categories: ordinary netizens, opinion leaders, and organizations (Chen et al., 2022). The participation of these groups in the “Rat Head and Duck Neck” incident is summarized in Table 1. It can be observed that ordinary netizens made up the majority (*n* = 21,150, 86.9%), followed by opinion leaders (*n* = 2,756, 11.3%) and organizations (*n* = 423, 1.7%). Despite opinion leaders and organizations representing only 11.3 and 1.7% of the total user base, their post volumes accounted for 14.0 and 2.4% of the total posts, respectively.

**Table 1:** Group participation in the “Rat Head and Duck Neck.”

**Category**	**Ordinary netizens**	**Opinion leaders**	**Organizations**
Number of users	21,150	2,756	423
Number of posts	24,567	4,119	712

### 2.3 Methods

**Figure 1:**
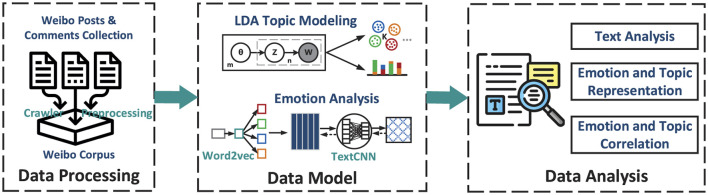
The technical framework of the research.

This paper employs computational methods such as topic analysis based on the LDA model, emotion analysis using machine learning with the TextCNN model, word frequency analysis, and other quantitative approaches. Additionally, it integrates qualitative methods like text analysis. The technical framework of this study is illustrated in Figure 1.

#### 2.3.1 LDA-based topic analysis

Latent Dirichlet Allocation (LDA) topic modeling is primarily used to identify hidden thematic information within large-scale document sets or corpora. It is essentially a three-level Bayesian probabilistic model that includes documents, topics, and terms, assuming that each document contains multiple topics and each topic is a probabilistic distribution over words. When constructing an LDA model, it is necessary to define the number of topics and partition the text corpus at varying granularities. Generally, the number of topics can vary depending on the quantity and complexity of the corpus. The optimal number of topics is typically determined using the Perplexity metric, which tends to decrease as the number of latent topics increases. The lower the perplexity, the better the probabilistic model distribution can predict the samples.

##### 2.3.1.1 Basic idea of LDA

**Figure 2:**
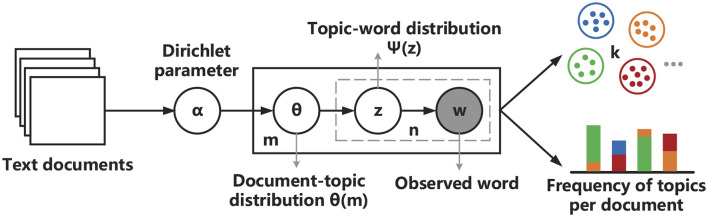
The basic idea of LDA topic modeling.

The fundamental idea behind LDA document generation is as follows: starting from a document, a topic is selected with a certain probability, and then a word is chosen from the selected topic with a certain probability. This process is iteratively repeated to generate enough words to form a document. The principle of the LDA topic model is exhibited in Figure 2. The specific process of executing the LDA model is as follows:

- Assume there are *m* documents to extract topics from, and the number of topics is *k*.
- For each document *m*, sample the document-topic probability distribution θ_*m*_ from a Dirichlet distribution with parameter α.
- For each word in document *m*, sample a topic *z* from the topic distribution θ_*m*_.
- For each topic *z* assigned to a word, sample a word *w* from the topic-word probability distribution ψ_*z*_.

##### 2.3.1.2 Execution in our case

**Figure 3:**
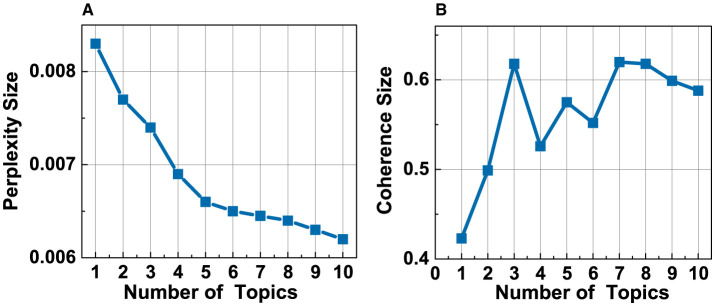
The optimal number for the initial stage. **(A)** Topic perplexity. **(B)** Topic coherence.

This study uses the gensim module in Python to build the LDA topic model, treating each Weibo post as a document and training the model on texts from different stages of the event. Since LDA is an unsupervised learning model, the number of topics needs to be determined manually, which significantly impacts the model's performance. The optimal number of topics is typically chosen based on two key criteria: perplexity and coherence. Perplexity generally decreases as the number of topics increases, but an excessively large number of topics may reduce the interpretability of the topics. Therefore, the optimal number is usually chosen when perplexity has decreased substantially but remains relatively low. Coherence, on the other hand, measures the internal consistency of words within a topic, indicating its interpretability. A higher coherence value suggests better topic consistency and clarity. For example, when determining the optimal number of topics for the initial stage, Figure 3 shows that perplexity drops sharply as the number of topics increases from 1 to 5, and then decreases more slowly. When the number of topics is 5, the coherence value reaches a relatively high level, making 5 the optimal choice for the initial stage. Based on this, the topics and their keywords for the initial stage were identified. However, perplexity and coherence are not the only factors considered. The topic analysis results are also adjusted based on communication theory perspectives. As a result, some overlapping topics were merged, and the final optimal number of topics for the initial stage was adjusted to 4. Following the same process, the optimal number of topics for the outbreak stage, first decline stage, second growth stage, second decline stage, and calm stage were determined to be 5, 7, 3, 3, and 1, respectively.

#### 2.3.2 Machine learning-based emotion analysis

**Figure 4:**
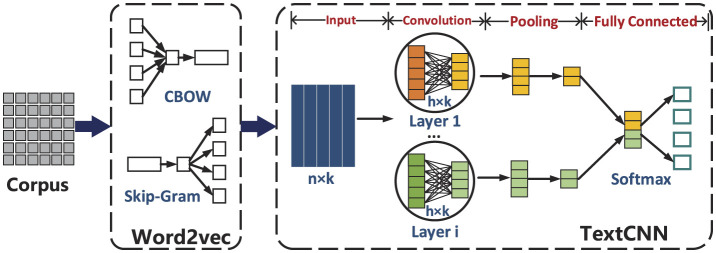
The basic idea of Word2vec and TextCNN.

To conduct the emotion analysis, this study first combines basic emotion categories and the composition of emotions in online discourse to apply Ekman's emotional classification standard (Ekman et al., 2013), while also adding satire as a compound emotion. The focus is on examining seven emotions in online public opinion during emergencies: anger, disgust, satire, fear, sadness, surprise, and happiness. It is important to note that in the practical implementation of emotion analysis, if any emotion has a low proportion, emotions with less than 5% representation are grouped as others and not analyzed in detail. Additionally, since happiness is the only positive emotion in Ekman's emotional classification, this study adopts the views of Cherry and VandenBos, considering happiness to encompass concepts such as joy, contentment, and satisfaction (Liu and Ding, 2020). After this, two machine learning models, Word2Vec and TextCNN, are used to identify emotional expressions in the text, as shown in Figure 4.

##### 2.3.2.1 Basic idea of Word2Vec model

The Word2Vec model, proposed by Mikolov et al. (2013), is a word embedding technique that transforms unstructured textual data into numerical tensors. It captures the proximity between two words in a sentence from two perspectives. First, if the meanings of the words are similar, their vectors will be close to each other, either in distance or angle. Second, the words are likely to co-occur in the same sentence, and swapping one word for another would not alter the sentence's meaning. Based on these principles, Word2Vec offers two primary training models: the Continuous Bag-of-Words (CBOW) model, which predicts a target word from its context, and the Skip-Gram model, which predicts the context of a word based on its vector. Both models essentially train a neural network using adjacent words in a sentence, with the goal of deriving network parameters that act as text features, rather than directly outputting features.

##### 2.3.2.2 Basic idea of TextCNN mode

The TextCNN neural network model is a variant of Convolutional Neural Networks (CNN), consisting of an input layer, convolutional layers, pooling layers, and a fully connected layer. The description and processing flow for each layer are as follows:

- Input layer: the function of this layer is to input data features into the convolutional neural network model and connect them to the next layer. The input layer is an *n*×*k* matrix, where *n* is the number of words in the sentence, and *k* is the dimension of the word embeddings. If *X*_*i*_ represents the word embedding for the *i*-th word in the text, the sentence can be represented as: *X*_*i*:*n*_ = [*X*_1_, *X*_2_, ..., *X*_*n*_].
- Convolutional layer: the purpose of this layer is to extract local features from the text. The convolutional layer consists of multiple convolution modules, each with a filter containing a set of neurons with fixed weights. The size of the filter is determined by the number of words it spans vertically and the dimension of the word embeddings. The filter computes relationships between adjacent words and extracts feature vectors from the sentence. Each filter generates one feature. The computation for each feature is given by:
  $$C_{h,i}=f\left(W_{h}\cdot X_{i:i+h-1}+b\right)$$
  where *f* is the ReLU activation function, *W*_*h*_ is the weight matrix of the convolutional kernel ($W_{h}\in {\mathbb{R}}^{h\times k}$), *b* is the bias, and *X*_*i*:*i*+*h*−1_ represents the word embeddings from the *i*-th to the (*i*+*h*−1)-th word in the sequence. The output of the convolution operation is *C*_*h, i*_.
- Pooling layer: this layer reduces the high-dimensional features extracted by the convolution layer. It uses abstraction techniques, such as max or average pooling, to lower the number of parameters and prevent overfitting, creating a new vector by concatenating the pooled values.
- Fully connected layer: the function of this layer is to integrate all the local features of the data and produce the final score, which is used for emotion analysis. The softmax function is then applied to output the classification probabilities.

##### 2.3.2.3 Execution in our case

This study follows a series of steps to preprocess and analyze Weibo text for emotion detection. These include data preprocessing, text vectorization with the Word2Vec model, and training the TextCNN neural network for emotion classification. We conducted parameter tuning based on prior research and empirical validation. The main parameters are set as follows: embedding dimension = 128, filter sizes = [3, 4, 5], number of filters per size = 100, dropout rate = 0.5, batch size = 64, and number of training epochs = 10. The Adam optimizer is used with a learning rate of 0.001. Below, we outline the methods used to prepare the data and evaluate the model's performance.

- Data preprocessing: the data preprocessing consists of the following steps: (1) Stopword removal and Chinese word segmentation: the text from Weibo is processed using regular expressions in Python and the Jieba natural language processing library to filter stopwords and segment the text. To optimize the segmentation process, specific terms related to the “Rat Head and Duck Neck” incident, such as pointing the rat as a duck, are added to the user-defined dictionary and incorporated into the segmentation system. (2) Emotion labeling: 30% of the collected data samples are randomly selected for emotion classification. Each Weibo post is assigned an emotion category based on the classification standard adopted in this study. It should be noted that since the proportions of sadness and surprise are both less than 5% in the labeled samples, they are categorized as Other.
- Text vectorization: after data preprocessing, the text is vectorized using the Word2vec model, where the words in the text are represented as word vectors that capture semantic relationships.
- Training and validation: (1) TextCNN training: the process involves feeding data into the model, performing convolution, pooling, and fully connected operations. (2) Model performance evaluation: the performance of the TextCNN model is evaluated using three metrics: Area Under the Curve (AUC), Precision, Recall, and F-measure. The values obtained are 0.89, 0.87, 0.85, and 0.84, respectively, demonstrating that this method is suitable for emotion analysis of Weibo text.

## 3 Results

In this section, we first present the development trajectory of the “Rat Head and Duck Neck” online public opinion incident. We then characterize the emotional and topic representations of the incident through topic analysis using the LDA model and emotion analysis with the TextCNN model. Finally, we explore the correlation between topics and emotions in online public opinion during emergencies.

### 3.1 Development trajectory of the “Rat Head and Duck Neck” online public opinion event

**Figure 5:**
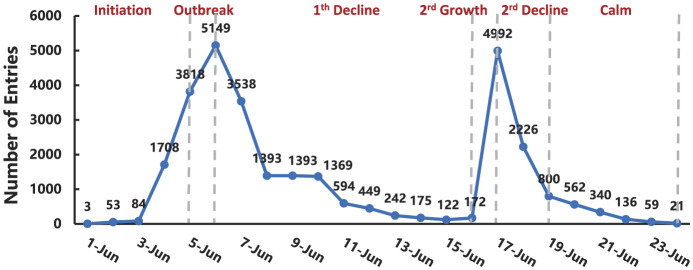
The development trajectory of the “Rat Head and Duck Neck” online public opinion incident.

Figure 5 illustrates the evolution of the “Rat Head and Duck Neck” incident on Chinese social media. The trajectory of public opinion exhibits a cyclical pattern, with multiple peaks and troughs that display distinct stage characteristics. Among these, June 6 and June 17 mark the two most prominent peaks of attention, with corresponding troughs on June 16 and June 19. The division of this event's developmental stages draws on the basic framework of crisis lifecycle theory (Coombs et al., 2019) and combines logical event nodes with empirical patterns in data fluctuation. Given the dissemination characteristics of the incident, the two peaks correspond to two pivotal moments: the first involves the heated public debate over the nature of the foreign object, and the second involves the release of the official investigation results. This dual-peak structure reveals a compound rhythm in the crisis's diffusion. Based on this logic and the features shown in Figure 5, the “Rat Head and Duck Neck” incident is divided into six stages: Initial stage (June 1–4), Outbreak stage (June 5–6), First decline stage (June 7–16), Second growth stage (June 17), Second decline stage (June 18–19), and Calm stage (June 20–24). An analysis of Weibo data during the two peaks indicates that the first peak was mainly driven by the intensifying debate over whether the object was a rat's head or a duck's neck, highlighting the tension between official narratives and public perception. The second peak emerged after the joint investigation team released its findings, which revealed the truth and triggered widespread public discussion. Overall, the themes of public discourse shifted from fragmentation to convergence, and from suspicion to reason.

### 3.2 The emotional and topic representations of online public opinion during emergencies

Online public opinion during emergencies encompasses both information flow and emotional flow, with topics and emotions jointly constructing the social representation of the incident. Accordingly, the composition and evolution of the topics and emotions related to the “Rat Head and Duck Neck” incident are analyzed.

#### 3.2.1 Composition and evolution of emotions for online public opinion in emergencies

This paper uses the emotion analysis method based on the TextCNN model (detailed in Section 2.3.2) to identify and analyze the emotions expressed in Weibo related to the “Rat Head and Duck Neck” incident.

(1) Emotional Tone of the “Rat Head and Duck Neck” Incident

Disgust, anger, and satire together form the emotional tone of the online public opinion surrounding the “Rat Head and Duck Neck” incident.

**Figure 6:**
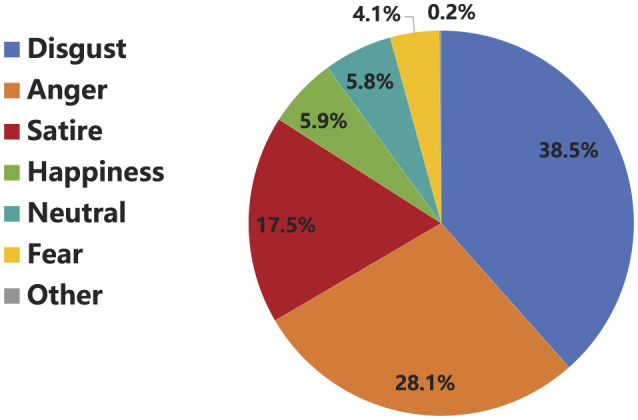
The emotion construction of the “Rat Head and Duck Neck” Incident.

Overall emotional composition: during the “Rat Head and Duck Neck” incident, aside from neutral (5.8%), the six emotions that appeared in the related Weibo, ranked from highest to lowest frequency, are: disgust, anger, satire, happiness, fear, and others, as shown in Figure 6. Among these, disgust is the dominant emotion in the online public opinion surrounding the incident, accounting for 38.5%. This is followed by anger (28.1%) and satire (17.5%). The remaining emotions, happiness and fear, appear less frequently, with their proportions ranging from 4.1 to 5.9%. Overall, the emotional tone of the “Rat Head and Duck Neck” incident's online public opinion is largely negative. Although there are emotions of happiness and humor-infused satire, they are not the predominant emotions.

Dominance of disgust and anger: according to the Integrated Crisis Mapping (ICM) theory (Jin et al., 2007), anger, fear, anxiety, and sadness are the primary emotions in crisis communication. Based on the ICM model, which categorizes the “Rat Head and Duck Neck” incident as a responsibility-related crisis in the first quadrant, anger and anxiety are expected to be the dominant emotions. However, this study finds that disgust and anger were the most prevalent emotions in Weibo discussions during the incident, deviating from the ICM theory. This discrepancy likely arises because the ICM theory was developed in a Western context and lacks localized application in China. Previous studies on crisis events related to responsibility in China have also confirmed disgust and anger as dominant emotions (Liu and Ding, 2020), consistent with our findings.

Further analysis reveals the following reasons for the dominance of disgust and anger in this case: First, in terms of crisis attribution, Jin et al. (2014) categorized disgust and anger as emotions linked to the responsible parties in a crisis. The key stakeholders in the “Rat Head and Duck Neck” incident include Nanchang Jinghe Catering Management Co., Jiangxi Industry Polytechnic College, and local market supervision authorities. As official responses from the school and government were evasive and misleading, the crisis evolved from a food safety issue to a public opinion crisis. Consequently, public emotions shifted from targeting businesses to government agencies and institutions, triggering a trust crisis. Second, the public in this case consisted largely of social media users, with ordinary netizens making up 86.9% of the group. Previous research on social media public opinion has shown that emotions like anger and disgust are commonly expressed in online crises (Liu and Ding, 2020). Finally, the event itself—centered on food safety issues affecting students and the abuse of public power—shocked the public's sense of morality and violated their expectations of fairness and justice, triggering widespread anger. Meanwhile, the public's dissatisfaction with the ambiguous responses and the ongoing debate over the nature of the foreign object led to growing disgust.

Prominence of satire: under the influence of multiple factors, satire emerged as a key emotional response in the “Rat Head and Duck Neck” incident. First, from a technical perspective, the grassroots nature, interactivity, and timeliness of the Weibo platform facilitated communication among netizens, enabling the rapid spread of satirical emotions. In the current internet culture, where entertainment and parody are prevalent, netizens harnessed satire to reflect social realities and raise public concerns. Second, from an ecological perspective, due to limitations in netizen literacy and regulatory standards, some netizens preferred to express their emotions in a more subtle and indirect manner, rather than resorting to blunt or harsh criticisms. Finally, from a social perspective, the ongoing social transformation in China has fostered dual emotional traits among the public: a strong desire for fairness and justice, and a tendency to mock the powerful and condemn corruption (Yang, 2009). In the “Rat Head and Duck Neck” incident, netizens used satire to express a variety of demands, including protecting students' rights, ensuring food safety, holding government bodies accountable for regulatory failures, and addressing conflicts of interest among responsible parties. Satire thus became a crucial outlet for the personalized expression of their opinions.

(2) Emotion evolution of the “Rat Head and Duck Neck” incident

**Figure 7:**
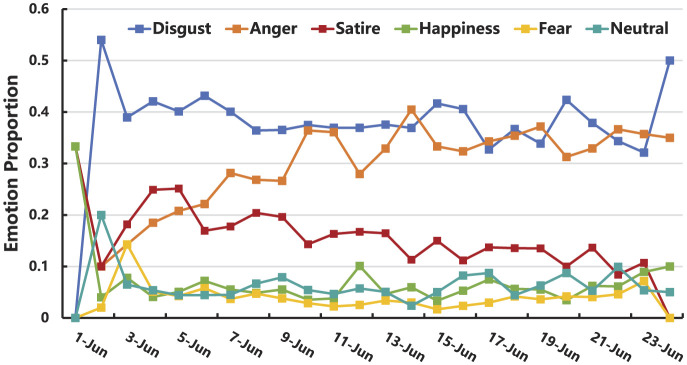
The variation in the proportion of emotions over time.

As shown in Figure 7, from June 1 to June 24, 2023, the emotional distribution of Weibo posts related to the “Rat Head and Duck Neck” incident fluctuated over time. Overall, this period can be divided into three distinct phases:

Stage 1 (June 1–6, 2023): during this phase, disgust dominates, with anger and satire showing an upward fluctuation. On June 1, news broke that a “rat head” was suspected in the cafeteria food at Jiangxi Industry Polytechnic College. The college and regulatory authorities responded, claiming the object was a duck neck, triggering a rat vs. duck debate on Weibo. Netizens experienced fear from the disturbing visuals, peaking on June 3, while disgust grew due to the ambiguity surrounding whether the object was a rat or a duck. Despite multiple investigations, the authorities provided only a vague response—it's just a duck neck. Amid growing skepticism, the public's anger began to intensify. Conspiracy theories emerged, such as swapping out samples before sending them for testing. Satirical content, such as memes and cartoons like “calling a rat a duck” flourished. On June 6, revelations about the university's media director organizing efforts to control the online discussion, combined with experts suggesting the object was likely a rat head, further fueled public outrage and distrust in the authorities.

Stage 2 (June 6–11, 2023): this phase is marked by a dominance of disgust, with a fluctuating rise in anger and a decline in satire. The “rat vs. duck” debate had lasted nearly a week, and netizens began to voice frustration, questioning, “In the age of advanced technology, how can it be so hard to determine if it's a rat or a duck?” This reflected growing irritation at the unresolved, seemingly simple issue. On June 8, a feedback group revealed that incidents of foreign objects in cafeteria food were common, and a new report surfaced about a student finding a large caterpillar in the same cafeteria, reigniting public concern over food safety and intensifying doubts about regulatory oversight. Consequently, anger continued to rise. Meanwhile, jokes and satirical content labeling the caterpillar as “cordyceps” or “green peppers” emerged, but their popularity remained below that of the original “rat head and duck neck” controversy, leading to a decline in satire. On June 10, the Jiangxi government formed a joint investigation team, and accusations of mishandling the situation grew louder. By this time, the proportion of anger had risen to the point where it intersected with disgust.

Stage 3 (June 11–24, 2023): in this phase, disgust and anger continued to intertwine, while the intensity of satire gradually declined. This phase marked the latter part of the first decline, the second outbreak, the second decline, and the period of calm for the “Rat Head and Duck Neck” incident. After the formation of a joint investigation team, the key stakeholders entered a calm period, and the public shifted to a state of waiting for the investigation results. During this time, online discussions were filled with reflections on the event and concerns about it being unresolved, leading to a dominance of anger and disgust in public emotions. Additionally, due to creative expressions related to the “Rat Head and Duck Neck” incident and other food safety concerns at the implicated cafeteria, happiness peaked on June 12. On June 17, when the investigation team confirmed that the foreign object in the meal was a rat head, netizens were pleased that the truth had come to light, and happiness reached its second peak. However, anger over the lack of accountability persisted, with calls for stricter food safety regulations and punishment for the responsible parties. Meanwhile, fear of an imbalance of power—fearing the loss of control over personal power and interests—led to a rise in fear, which peaked from June 17 to June 23, reaching its second peak on June 23.

#### 3.2.2 Composition and evolution of topics for online public opinion in emergencies

This paper applies topic modeling based on the LDA method (detailed in Section 2.3.1) to identify and analyze the topics in Weibo related to the “Rat Head and Duck Neck” incident.

(1) Topic composition of the “Rat Head and Duck Neck” incident

The development of the “Rat Head and Duck Neck” incident can be divided into the following stages: the initial stage, outbreak stage, first decline stage, second growth stage, second decline stage, and calm stage, exhibiting a bimodal evolution pattern, as shown in Figure 5. The corresponding Weibo discussion topics for each stage are outlined in Table 2.

**Table 2:** Topics on different stages of the “Rat Head and Duck Neck” incident.

**Phase**	**Topic number**	**Topic name**	**Topic keywords**
Initial	Topic I-1	Jiangxi Industry Polytechnic College canteen allegedly contained a rat head	Rat Head, Duck Neck, Video, Jiangxi, Foreign Object, Food, Canteen
	Topic I-2	Jiangxi Industry Polytechnic College releases situation report	Teeth, Duck Meat, Inspection, Confirmation, Jiangxi Industry Polytechnic College, Official
	Topic I-3	Nanchang Market Supervision Bureau confirms the foreign object is duck neck	Calling a Deer a Horse, Calling a Rat a Duck, Market, Supervision, Bureau
	Topic I-4	Netizens accuse Jiangxi Industry Polytechnic College and Nanchang Market Supervision Bureau of lying	Bureau Chief, Lying, Leadership, Falsehood, Calling a Deer a Horse, Calling a Rat a Duck, Graduation Certificate
Outbreak	Topic II-1	Stock prices of Juewei Duck Neck, Zhou Hei Ya and others drop	Juewei, Zhou Hei Ya, duck neck, victim, stockholder, Food Safety
	Topic II-2	Animal expert claims the rat head is likely a duck neck; school allegedly manipulated network comments	Expert, disgusting, director, animal, comments, strange, absurd
	Topic II-3	Netizens create a catchphrase for the incident—“Calling a Deer a Horse”	Calling a deer a horse, calling a rat a duck, Zhao Gao, ancient and modern, mismatch
	Topic II-4	Netizens believe the response to the incident did not address key issues, insulting intelligence	Rat head duck neck, key issues, intelligence, confusion, falsehood
	Topic II-5	Jiangxi Industry Polytechnic College claims the rat head was confirmed as duck neck by the student	Student, invitation, inspection, filming, careful, spread
1st Decline	Topic III-1	Jiangxi Provincial Department of Education intervenes in the Rat Head and Duck Neck incident	Involvement, Education Department, Jiangxi Province, Department, Journalists
	Topic III-2	Netizens demand key evidence from relevant authorities	Simple, provide, key, choice, follow-up
	Topic III-3	Jiangxi Industry Polytechnic College allegedly served a large green caterpillar in the canteen	Pickled pepper, green caterpillar, graduation certificate, canteen, beans, Cordyceps
	Topic III-4	Large numbers of students outside Jiangxi Industry Polytechnic College taking takeout	Continuing fermentation, takeout, caterpillar, rat head, sanitation
	Topic III-5	Netizens question food safety supervision measures	Food, evidence, identification, cucumber, canteen, cold noodles, interests
	Topic III-6	Investigation on the rat head duck neck incident is ongoing	Duck neck, rat, canteen, expert, news, investigation, discovery
	Topic III-7	Jiangxi Province establishes joint investigation group for the rat head duck neck incident	Investigation group, market, joint, establishment, supervision
2rd Growth	Topic IV-1	Joint investigation group determines the foreign object is not duck neck	Determination, kitchen, public trust, inquiry, investigation result, surveillance, photo
	Topic IV-2	Jiangxi Industry Polytechnic College deletes previous statement	Situation, statement, calling a rat a duck, media, Zhao Gao, deception
	Topic IV-3	Post-incident accountability in the Rat Head Duck Neck incident	Bureau chief, operator, food safety, school, Hu Xijin, responsibility
2rd Decline	Topic V-1	Follow-up actions of companies involved in the rat head duck neck incident	Zhongkuai, canteen, catering, Juewei, government, injustice
	Topic V-2	Food safety in universities sparks concern	Food safety, operator, canteen, school, leadership, producer
	Topic V-3	Investigation group determines the foreign object is a rodent head	Duck neck, rat head, determination, rodent, foreign object
Calm	Topic VI-1	Official credibility gap difficult to bridge	Credibility, impact, city, government, behind, interests

(2) Topic evolution of the “Rat Head and Duck Neck” incident

Through topic analysis and classification of Weibo related to the “Rat Head and Duck Neck” incident, this paper identifies the focal points of public attention and the evolution of public opinion throughout the incident's development. The 23 topics identified in the study can be broadly categorized into six major groups:

- Event development: Topic I-1, Topic II-2, Topic III-1, Topic III-6, Topic III-7, Topic IV-1, Topic IV-2, Topic IV-3, Topic V-3
- Responses: Topic I-2, Topic I-3, Topic II-5
- Doubts: Topic I-4, Topic II-3, Topic II-4, Topic III-2, Topic III-5
- Corporate turmoil: Topic II-1, Topic V-1
- Derivative issues: Topic III-3, Topic III-4
- Event impact: Topic V-2, Topic VI-1

**Figure 8:**
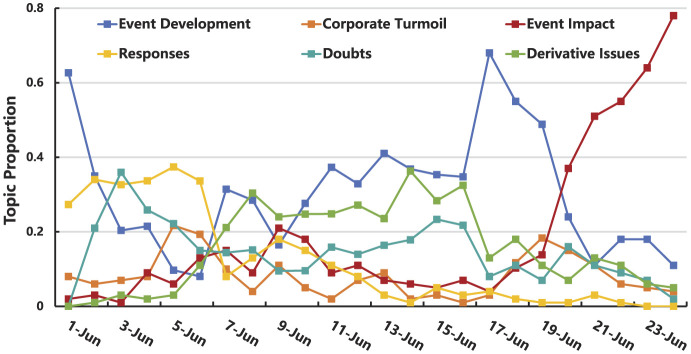
The variation in the proportion of topics over time.

In general, except for the second decline and calm stages, where the heat of the topics largely stabilizes, the topics in other stages show significant fluctuations in intensity. Netizens closely followed the development of the incident through the Weibo platform. From the perspective of the changing topic proportions over time, as shown in Figure 8, the Event Impact category shows increasing heat, while Responses tend to decrease. Derivative issues, Corporate Turmoil, and Doubts exhibit fluctuating trends in heat. Looking at the emphasis of topics in different stages: In the initial stage, the focus was mainly on Event Development, Doubts, and Responses from various parties. During the outbreak stage, the attention shifted to Event Development and Derivative Issues. In the first decline stage, topics related to Event Development, Derivative Issues, and Doubts gained significant attention. In the second growth stage, Event Development continued to be a major focus. Following this, the attention shifted primarily to the Event Impact.

### 3.3 The correlation between topics and emotions in online public opinion during emergencies

This section delves into the correlation mechanisms between topics and emotions in emergencies. It begins by integrating topic-emotion analyses to examine the evolution of online public opinion in emergencies. Following this, it explores how emotions construct online public opinion, focusing on both external manifestations and intrinsic dynamics.

#### 3.3.1 Evolution of online public opinion in emergencies based on topic-emotion integration

**Figure 9:**
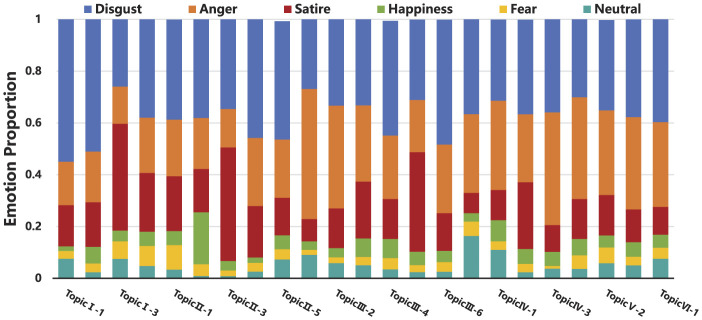
Emotion-topic proportion on different stages of the “Rat Head and Duck Neck” incident.

As shown in Figure 9, emotional analysis was conducted on Weibo from different stages of the “Rat Head and Duck Neck” incident's online public opinion development. From the overall lifecycle of public opinion evolution, the initial and outbreak stages were primarily dominated by disgust and satire, while later stages were primarily driven by disgust and anger. The reason lies in the unresolved “Rat Duck Mystery.” In the early stages of the event, the relevant parties spoke out and published the situation and investigation results. Netizens who questioned the official statements expressed their expectations for clarification with relatively mild and gentle emotions. However, the parties involved did not understand the key to preventing the continued development of negative public opinion. As a result, netizens switched to more direct and sharp emotional expressions, demanding the restoration of the facts and severe punishment for the responsible parties.

In the Weibo topic discussions during different stages of the “Rat Head and Duck Neck” incident, most topics are underpinned by disgust, but a few are framed with satire or anger. Specifically, Topic I-3 (Nanchang Market Supervision Bureau confirms the foreign object is duck neck), Topic II-3 (Netizens create a catchphrase for the incident—“Calling a Deer a Horse”), and Topic III-5 (Netizens questioning food safety supervision measures) prominently feature satire, all of which reflect doubt toward the government's discourse and actions, highlighting the trust topic. Topic III-1 (Jiangxi Provincial Department of Education intervenes in the Rat Head and Duck Neck incident), Topic III-2 (Netizens demand key evidence from relevant authorities), and Topic IV-3 (Post-incident accountability in the Rat Head and Duck Neck incident) prominently feature anger, each focusing on demands for clarification of the developments, responses to the truth, and punishment for the responsible parties, highlighting the responsibility topic.

#### 3.3.2 The constructive role of emotions in online public opinion during emergencies

The emotional distribution of different topics in various stages of the evolution is merely an external manifestation of the constructive role that emotions play in shaping online public opinion. Under the influence of emotions, specific emotional orientations often underpin the discussions of different topics (Nussbaum, 2003). Therefore, through the analysis of content with varying emotional intentionality, we will examine the content organization principles under the influence of emotions.

(1) The intentional content of disgust emotions in online public opinion

A word frequency analysis of Weibo expressing disgust emotions reveals the top 20 most frequent words, as shown in Table 3. High-frequency terms such as “Duck Neck,” “Rat,” and “Rat Head” indicate that expressions of disgust are primarily centered on the event itself, without extending to discussions of food safety and accountability behind the incident. This aligns with the findings of Horberg et al. (2009), which suggest that disgust emotions only influence moral judgments in purity domains, not in judgments related to justice or fairness. In terms of emotional expression, disgust is conveyed more moderately and rationally compared to anger.

**Table 3:** Top 20 high-frequency words related to disgust emotion in the “Rat Head and Duck Neck” incident.

**Rank**	**High-frequency words**	**Word Frequency**
1	Duck neck	1,800
2	Rat	1,274
3	Rat head	565
4	Video	324
5	Student	371
6	School	316
7	Canteen	309
8	Teeth	234
9	Bureau chief	221
10	Object	211
11	Sent for inspection	203
12	Testing	195
13	Identification	193
14	Duck	192
15	Indeed	185
16	Certainly	159
17	Investigation	137
18	Eyes	137
19	Picture	137
20	Disgust	210

In the “Rat Head Duck Neck” incident, the disgust emotions expressed by netizens include both core disgust and moral disgust. Core disgust primarily relates to the potential disease-spreading rats, which, as carriers of infectious viruses, become the stimuli for core disgust. Moral disgust, on the other hand, is expressed in reaction to the act of lying, a violation of moral standards. When key information remains unknown and crucial questions unanswered, netizens, based on personal life experience and the principle of seeing is believing, judge the foreign object as a rat head and believe the responsible parties in the incident are lying, violating the moral norms of integrity, thus intensifying their disgust emotions.

(2) The intentional content of anger in online public opinion

A word frequency analysis was conducted on Weibo posts expressing anger, and the top 20 high-frequency words are presented in Table 4. Terms such as “Public Credibility,” “Food Safety,” “Calling a Rat a Duck,” “Lying,” and “Outrageous” reflect the underlying reasons for netizens' anger. References to “Bureau Chief,” “Department,” “Government,” “Jiangxi,” “Official,” and “Leader” indicate the primary targets of public dissatisfaction, while words such as “Severe Punishment,” “Investigation,” and “Admission” highlight the demands articulated by netizens. The expression of anger is framed within a binary narrative structure opposing official authorities and ordinary citizens. Netizens frequently employed exclamation marks, question marks, and strong language to convey intense anger, skepticism, and dissatisfaction.

**Table 4:** Top 20 high-frequency words related to anger in the “Rat Head and Duck Neck” incident.

**Rank**	**High-frequency words**	**Word frequency**
1	Public credibility	895
2	Bureau chief	400
3	Food safety	498
4	Market	280
5	Department	278
6	Calling a rat a duck	268
7	Investigation	249
8	Society	242
9	Government	240
10	Lying	376
11	Official	201
12	Calling a deer a horse	193
13	Jiangxi	190
14	Severe punishment	198
15	Simple	168
16	Outrageous	198
17	Object	167
18	Admission	164
19	Leader	163
20	Identification	158

Although anger is typically viewed as a negative emotion with potential social drawbacks, its positive value cannot be overlooked. The cognitive factors behind anger are precisely what give it its value. In the context of the internet, anger, more than other emotions, is capable of pushing societal order forward and promoting its improvement, driving rational public opinion. By analyzing the intentional content of anger, this paper explores the moral grammar and principles of justice underlying the emotion. According to Table 4, “Food Safety” and “Public Credibility” are the primary sources of anger for netizens in the “Rat Head and Duck Neck” incident. “Food safety” relates to issues of rights violations, with the underlying moral grammar being the inviolability of individual rights. The issue of food safety, especially for students, easily evokes moral shock, making anger the dominant emotion, as many netizens raise the call, “Food safety cannot be ignored.” “Public Credibility” pertains to the abuse of power, with the underlying moral grammar being the legitimacy of public power usage. Due to the sensitivity surrounding the supervision of power, the “Rat Head and Duck Neck” incident quickly resonated with netizens after spreading online, and netizens strongly condemned the government's dereliction of duty. Overall, in the “Rat Head and Duck Neck” incident's online public opinion, netizens used anger as a moral critique, expressing their dissatisfaction with the violation of public rights and the improper use of public power, advocating for the protection of public interests, the regulation of political behavior, and thus maintaining the moral order and sense of justice they hold.

(3) Intentional content of satire in online public opinion

A frequency analysis was also performed on Weibo comments expressing satirical emotions. After excluding meaningless words, the top 20 high-frequency terms were identified, as shown in Table 5. Notably, terms such as “Calling a Rat a Duck” appear humorous at first glance but actually convey a complex mix of emotions, including anger, resentment, and skepticism. These expressions serve to alleviate public anger over the collapse of trust and simultaneously articulate dissatisfaction with the school's disregard for students' rights and the government's negligence regarding food safety. The phrase “Calling a Rat a Duck” is derived from the idiom “Calling a Deer a Horse” in Records of the Grand Historian. In the negative perceptions of netizens, the main authority figure in this case, the “Bureau Chief,” is likened to “Zhao Gao,” while the market supervision bureau's actions of ignoring the truth and distorting facts are described as “Calling a Rat a Duck.” This newly coined phrase employs a parody strategy, which emerges as the primary method by which netizens express satirical emotions in this incident.

**Table 5:** Top 20 high-frequency words related to satire in the “Rat Head and Duck Neck” incident.

**Rank**	**High-frequency words**	**Word frequency**
1	Calling a rat a duck	757
2	Calling a deer a horse	633
3	Zhao Gao	281
4	Ancient and modern	237
5	Humble rat like me	231
6	Now and then	203
7	Graduation certificate	189
8	Student	173
9	School	173
10	Bureau chief	118
11	Humble rat	101
12	New species	101
13	Repeated	98
14	Pickled pepper	89
15	Obviously	79
16	Idiom	77
17	Donald duck	71
18	Mickey mouse	70
19	Cordyceps	68
20	Name	67

Satire, as a combination of anger and humor, shares a similar moral framework with anger, involving the inviolability of individual rights and the legitimacy of public power use. The inviolability of individual rights encompasses food safety protection and freedom of speech, while the legitimacy of public power use involves issues such as “Calling a Deer a Horse” and “Official-Business Collusion.” In contrast to anger, which focuses on “Food Safety” and “Public Credibility,” the moral grammar underlying satirical emotions is more multifaceted. Overall, expressions of satirical emotions exhibit the following characteristics: Fixed cognitive patterns: the discourse of satirical emotions mirrors that of anger, with a binary narrative structure that targets the conflicts between the government, schools, and the public, opposing official discourse. Rich emotional layers: satire possess a dual emotional quality—outward joy, inward rebellion. Naturally, depending on the content of the comments, the dual emotional aspects may vary in prominence. Multilayered semantic connotations: compared to the direct criticisms associated with disgust or anger, the expression of satire is more indirect, with words often carrying multiple interpretations. One-sided information expression: in contrast to reasoned analysis, satire expressions tend to focus on questioning specific aspects of the issue, often omitting the broader context and employing simple, low-logic expressions.

## 4 Awakening, condensation, and sedimentation: the evolutionary mechanism of online emotions

The emergence and evolution of online public opinion during emergencies is not merely a process of information transmission but also one of psychological processing and social meaning construction. As a bridge between the individual and the collective, social representation transforms abstract information into stable frameworks of meaning, guiding public understanding and response. The dynamic construction of such representations is driven by psychosocial anchoring, which integrates individual cognition and emotional response. Emotions serve as a key mediating force between anchoring and meaning construction, driving the formation and transformation of public discourse in times of crisis. Based on the proposed anchoring-mediation-representation model, this section reveals the phased characteristics of online emotional evolution in the “Rat Head and Duck Neck” incident. The confrontational, satirical, and moralized emotional expressions observed throughout the incident reflect a complete pathway from emotionally driven anchoring to symbolic construction.

### 4.1 Awakening: emotion activation anchored in collective memory

The awakening of emotion is not accidental but rooted in the latent influence of collective memory. When confronted with highly uncertain events, individuals instinctively seek familiar cognitive references to reduce processing burden. Collective memory, as a shared experiential system within a particular social group, becomes the primary source of psychosocial anchoring. It functions as a repository of emotional schemas that are activated by current events, thereby triggering emotional responses associated with similar past incidents. These reactivated emotions provide the emotional framework for public interpretation and judgment. In the early phase of the “Rat Head and Duck Neck” incident, disgust emerged as the dominant emotion. High-frequency terms such as “Duck Neck,” “Rat,” and “Rat Head” suggest that the public initially framed the event as a food safety crisis. As a typical negative emotion in such contexts, disgust functions as a physiological defense mechanism aimed at avoiding contamination or harm. This is consistent with the identified Topic I-1 (Jiangxi Industry Polytechnic College canteen allegedly contained a rat head), which aligns closely with the prevalence of disgust. As the incident unfolded, the ambiguous official response, claiming the object was a duck neck, provoked intense skepticism. In the absence of key information, the public shifted its anchoring to previous high-profile food safety scandals such as “Melamine Contamination Crisis,” “Gutter Oil,” and “Lean Meat Powder,” as well as to narratives of corporate-government collusion. These collective memories shaped the public's understanding of the current event. Many netizens concluded that the object was indeed a rat head and framed the response as deceitful and complicit. Emotions such as anger and satire were thus awakened, and expressions of confrontation and mockery began to proliferate. Drawing on these shared memory codes, netizens endowed their responses with richer symbolic meaning and intensified critical force.

### 4.2 Condensation: emotional resonance and symbolization driven by shared values

Individual emotions can be transformed into powerful social emotions through the driving force of shared values. While collective memory provides the material for emotional awakening, shared values legitimize these emotions and serve as the foundation of psychosocial anchoring. In the context of online interaction, emotional expression is not only a means of defending rights and seeking justice but also a manifestation of value judgments and collective identity. Through expressive interactions, individuals converge into temporary communities that coalesce around specific concerns. In the “Rat Head and Duck Neck” incident, dispersed individuals quickly converged, and intense emotions spread across this ephemeral online community. Anger was reflected in high-frequency words such as “Public Credibility,” “Bureau Chief,” and “Food Safety,” while satire was expressed through phrases like “Calling a Rat a Duck,” “Calling a Deer a Horse,” and “Zhao Gao.” The contrast between “Rat Head” and “Duck Neck” took on symbolic significance. For example, “Calling a Rat a Duck” referenced a classical idiom to satirize the distortion of truth by official authority. Shared values imbued these symbols with moral legitimacy and emotional coherence, turning them into core resources for collective expression. It was the public's adherence to values such as integrity and fairness that gave rise to widespread resonance and the symbolization of anger and satire. These emotions were transformed into targeted moral sentiments, generating immense public pressure and further reinforcing the agenda-setting power of emotionally driven discourse.

### 4.3 Sedimentation: social representation and the formation of latent public opinion currents

The resolution of a public crisis does not signal the dissipation of associated emotions. Strong emotions tend to sediment over time, and the cognitive, emotional associations formed during information processing persist within the collective psyche. Social representation theory emphasizes that meanings widely shared within a group are gradually solidified through continuous circulation and become part of a stable cognitive framework. In the case of the “Rat Head and Duck Neck” incident, core emotional reactions, including skepticism toward public credibility, dissatisfaction with food safety oversight, and anxiety over systemic vulnerabilities, did not dissipate with the incident's closure. Instead, they were embedded in the public's emotional memory and value system, forming a baseline emotional tone. This tone accumulates over time in media environments and social interactions, ultimately forming latent currents of public opinion. This process of emotional sedimentation reflects the objectification mechanism in social representation. Specific emotion-symbol combinations, such as “Calling a Rat a Duck,” encapsulate the public's distrust and satirical criticism of authority. These combinations, once widely disseminated and collectively endorsed, become shared social representations. They serve as cognitive anchors in interpreting similar events, becoming embedded in collective memory. In future incidents involving government accountability or food safety, the public is likely to reactivate cognitive-emotional schemas rooted in the “Rat Head and Duck Neck” case. This preconditioned response, a product of psychosocial anchoring, will significantly influence the trajectory of subsequent public discourse and meaning construction. Even though the investigation clarified the facts, the sedimented emotions of disappointment and distrust have already crystallized into shared representations of public credibility and food safety, becoming enduring emotional resources in the collective interpretive system.

## 5 Discussion

This study investigates the “Rat Head and Duck Neck” incident, a widely discussed and socially significant food safety case, using Weibo as the primary data source. Grounded in social representation theory and psychosocial anchoring theory, and applying LDA-based topic modeling, TextCNN-based sentiment analysis, and qualitative textual analysis, we systematically explore the co-evolution mechanism between topics and emotions in online public opinion during emergencies. This study not only broadens the analytical perspective on public opinion dynamics but also contributes new theoretical and empirical insights into the mediating role of emotion in cognitive anchoring and social meaning construction.

First, the emotional tone of the “Rat Head and Duck Neck” event was dominated by disgust, anger, and satire. Prior studies have shown that anger and disgust are among the most representative negative emotions in responsibility-type crisis events, often targeting those deemed responsible (Coombs, 2007; Coombs and Tachkova, 2019). These emotions typically reflect the moral-critical and confrontational nature of public discourse during crises. In addition to these, this study finds satire to be particularly salient. As a hybrid emotional expression combining humor and resistance (Wu and Li, 2016; Chen and Ding, 2018), satire was widely employed in this incident. Netizens adapted the historical idiom “Calling a Deer a Horse” into “Calling a Rat a Duck” as a form of mocking the official response. This reframing not only reinforced the public's sense of absurdity regarding the incident but also transformed personal discontent into a collectively shared form of resistant humor. This finding enriches our understanding of emotional diversity in crisis communication and reveals the symbolic power of satire in contexts of trust breakdown.

Secondly, in terms of topic evolution mechanisms, public opinion in emergency events typically follows a linear progression from fact-checking to responsibility attribution and institutional reflection. While the “Rat Head and Duck Neck” incident began as a food safety controversy, public discussion quickly shifted from questioning the factual accuracy of the event to critiquing the loss of institutional trust, revealing a critical turn from specific events to the macro-level governance structure. This evolution aligns closely with the crisis lifecycle model (Sturges, 1994), which describes the shift in crisis topics from factual concerns to value-oriented debates. The metaphorical expression “Calling a Rat a Duck” in the incident, with its strong symbolic connotation, not only served as a tool for expressing dissatisfaction with the official response but also became a symbolic vehicle for the public's perception of power dereliction and media manipulation. Therefore, the core issue of the “Rat Head and Duck Neck” incident is not merely about assigning responsibility but reflects a structural distrust of governance systems perceived as unable to effectively constrain power. Previous studies have pointed out that the Chinese public's perception of food safety risks differs from the Western emphasis on systemic risks. It tends to attribute such risks to the individual moral failure of producers or vendors, rather than merely to technical deficiencies or regulatory system weaknesses (Yan, 2012; Yu et al., 2024). This cognitive tendency to attribute systemic issues to individual moral failings further exacerbates the trust gap between the government and the public and amplifies the sense of unfair distribution of food safety risks among different social groups. Moral attribution becomes the logical premise for institutional critique, where individual moral violations easily lead to imagined collusion between officials and businesses, ultimately resulting in a fundamental doubt regarding institutional trust. This reveals the deep interactive logic between moral attribution and institutional critique in Chinese public opinion on food safety.

Finally, in terms of emotional evolution, this study proposes a three-stage mechanism model: anchoring–mediation-representation, which offers a new perspective for understanding the psychological mechanisms underlying the evolution of public opinion during emergencies. Emotion is not only a fundamental component of social representation but also a key driving force in its construction and transformation. The emotional progression in crises typically follows a sequential path of awakening, condensation, and sedimentation. When faced with highly uncertain events, individuals tend to anchor new emotional experiences to memories to reduce cognitive load and form initial emotional judgments. Collective memory, as a pre-existing cognitive–emotional schema, provides the psychological basis for rapid emotional activation. Once externalized in social media spaces, individual emotions are amplified and replicated through the structure of online networks. Through symbolic expressions, fragmented individual emotions are gradually transformed into coherent and goal-oriented collective moral emotions, fostering the formation of emotional communities around specific issues. Emotional condensation enables social resonance through shared values. Emotion–topic combinations that repeatedly appear and gain recognition during the dissemination process gradually solidify into stable interpretive frameworks and become embedded in the public subconscious. Through ongoing circulation and collective attribution of meaning, these emotional structures not only serve as the cognitive baseline for interpreting this particular event but also become pre-established resources for emotional responses and opinion formation in future similar crises. This mechanism model not only reveals the internal logic of how emotions evolved in the “Rat Head and Duck Neck” incident—from individual awakening to collective condensation and finally to structural sedimentation, but also highlights the emotion-driven nature of social representation. Emotion serves both as a constitutive element of representation and as a core force driving the structural transformation of public discourse. Thus, the model provides a novel theoretical lens and explanatory framework for understanding the interactive mechanisms among cognitive categorization, value resonance, and meaning construction in the context of public emergencies.

Despite the theoretical integration and empirical exploration undertaken in this study, several limitations remain. On one hand, the data source is limited to the Weibo platform. Although Weibo is highly representative in the context of online public opinion regarding public emergencies in China, the platform's discourse ecology and content moderation mechanisms may affect the comprehensiveness and diversity of discussions. Therefore, the external validity of the findings needs to be further evaluated and expanded in future research through cross-platform data validation. Although this study employed strategies such as early data collection and cross-referencing with information from other platforms to mitigate the risk of data bias, the impact of censorship interventions, such as content deletion, cannot be entirely ruled out. Future studies could incorporate additional data sources such as Zhihu, Douyin, and WeChat public accounts to provide a more comprehensive picture of the structure and dynamics of China's online public opinion environment. On the other hand, although this study adopts a combined strategy of quantitative computation and qualitative analysis, the identification of implicit emotions in complex texts still poses technical challenges. Subsequent research may consider incorporating deep semantic analysis and emotion computing models to further improve the accuracy of emotion detection and enhance the explanatory power of the underlying mechanisms.

In summary, through theoretical integration and empirical analysis, this study proposes a new explanatory framework for understanding the co-evolution of emotions and topics in the context of public emergencies. It also offers valuable empirical insights for further research on crisis communication, particularly regarding cultural contextual differences, emotional functionality, and social cognitive mechanisms.

## Data Availability

The original contributions presented in the study are included in the article/supplementary material, further inquiries can be directed to the corresponding authors.

## Footnotes

^1^Popular social media platform in China (https://weibo.com).

^2^ https://www.wechat.com

^3^ https://www.zhihu.com

^4^Baidu Index is a search trend analysis platform based on Baidu's user data in China.
